# Hematologic and Immunologic Overlap Between COVID-19 and Idiopathic Pulmonary Fibrosis

**DOI:** 10.3390/jcm14155229

**Published:** 2025-07-24

**Authors:** Gabriela Mara, Gheorghe Nini, Stefan Marian Frenț, Coralia Cotoraci

**Affiliations:** 1 Multidisciplinary Doctoral School, Vasile Goldis Western University of Arad, 310414 Arad, Romania; mara.gabriela@uvvg.ro; 2 Pneumology Department, Vasile Goldis Western University of Arad, 310414 Arad, Romania; nini.gheorghe@uvvg.ro; 3 Centre for Research and Innovation in Precision Medicine of Respiratory Diseases, Department of Pulmonology, University of Medicine and Pharmacy Timisoara, 300041 Timisoara, Romania; frentz.stefan@umft.ro; 4 Clinical Hematology Department, Vasile Goldis Western University of Arad, 310025 Arad, Romania

**Keywords:** idiopathic pulmonary fibrosis, COVID-19, cytokine storm, endothelial dysfunction, fibrosis, immune dysregulation, thrombosis

## Abstract

Idiopathic pulmonary fibrosis (IPF) is a progressive fibrosing lung disease characterized by chronic inflammation, vascular remodeling, and immune dysregulation. COVID-19, caused by SARS-CoV-2, shares several systemic immunohematologic disturbances with IPF, including cytokine storms, endothelial injury, and prothrombotic states. Unlike general comparisons of viral infections and chronic lung disease, this review offers a focused analysis of the shared hematologic and immunologic mechanisms between COVID-19 and IPF. Our aim is to better understand how SARS-CoV-2 infection may worsen disease progression in IPF and identify converging pathophysiological pathways that may inform clinical management. We conducted a narrative synthesis of the peer-reviewed literature from PubMed, Scopus, and Web of Science, focusing on clinical, experimental, and pathological studies addressing immune and coagulation abnormalities in both COVID-19 and IPF. Both diseases exhibit significant overlap in inflammatory and fibrotic signaling, particularly via the TGF-β, IL-6, and TNF-α pathways. COVID-19 amplifies coagulation disturbances and endothelial dysfunction already present in IPF, promoting microvascular thrombosis and acute exacerbations. Myeloid cell overactivation, impaired lymphocyte responses, and fibroblast proliferation are central to this shared pathophysiology. These synergistic mechanisms may accelerate fibrosis and increase mortality risk in IPF patients infected with SARS-CoV-2. This review proposes an integrative framework for understanding the hematologic and immunologic convergence of COVID-19 and IPF. Such insights are essential for refining therapeutic targets, improving prognostic stratification, and guiding early interventions in this high-risk population.

## 1. Introduction

The global emergence of coronavirus disease 2019 (COVID-19), caused by the severe acute respiratory syndrome coronavirus 2 (SARS-CoV-2), has significantly reshaped the landscape of modern medicine [1]. Since its initial identification in late 2019, the virus has demonstrated a diverse spectrum of clinical manifestations, ranging from asymptomatic infections to severe pneumonia, acute respiratory distress syndrome (ARDS), multiorgan failure, and death [2,3]. Beyond its pulmonary involvement, COVID-19 exhibits profound hematologic and immunologic dysregulation, including cytokine storm, hypercoagulability, lymphocyte depletion, and endothelial dysfunction [4,5]. Severe COVID-19 is frequently marked by lymphocyte depletion, increased neutrophil-to-lymphocyte ratio (NLR), platelet abnormalities, coagulopathy, and a hypercytokinemic state [6]. These systemic alterations have exposed new vulnerabilities in patients with pre-existing chronic lung diseases, particularly idiopathic pulmonary fibrosis (IPF) [7]. SARS-CoV-2 enters host cells via the angiotensin-converting enzyme 2 (ACE2) receptor, which is abundantly expressed on alveolar epithelial and endothelial cells [8]. Viral binding leads to ACE2 downregulation, disrupting the protective ACE2/Ang-(1–7)/Mas axis of the renin–angiotensin system [9]. This shift promotes increased angiotensin II signaling, driving vasoconstriction, inflammation, endothelial damage, and fibroblast activation. These effects are highly relevant in the context of idiopathic pulmonary fibrosis, where a pre-existing fibrotic and inflammatory environment may be further exacerbated by COVID-19 infection.

IPF is a chronic, progressive, and often fatal interstitial lung disease of unknown etiology, characterized by the relentless deposition of the extracellular matrix and architectural distortion of the lung parenchyma [10]. The disease predominantly affects older adults and is associated with comorbidities that further compound clinical outcomes [11]. IPF has a median survival of approximately three to five years from diagnosis, with limited therapeutic options beyond antifibrotic agents such as pirfenidone and nintedanib [12]. The vulnerability of IPF patients to respiratory infections is well recognized, and the introduction of a novel pathogen such as SARS-CoV-2 into this population raises significant concerns.

IPF patients represent a population at high risk of poor outcomes in the setting of COVID-19 [13]. This vulnerability compromised lung function and had systemic alterations that may exacerbate the severity of SARS-CoV-2 infection [14,15]. Among these alterations are underlying hematologic abnormalities and a predisposition to immune dysregulation and thromboembolic events. The interface between the hematological manifestations of COVID-19 and the pathophysiological milieu of IPF creates a “perfect storm,” wherein the two diseases may synergistically amplify inflammation, fibrosis, and coagulation abnormalities.

Unlike broader literature reviews that examine viral infections or chronic pulmonary diseases in general, this review offers a focused analysis of the hematologic and immunologic interplay specifically between COVID-19 and idiopathic pulmonary fibrosis (IPF). By emphasizing shared mechanisms such as endothelial dysfunction, immune dysregulation, and microvascular thrombosis, we aim to provide a mechanistic framework for understanding how SARS-CoV-2 infection may exacerbate fibrosis progression and clinical deterioration in this vulnerable patient population.

## 2. Materials and Methods

This review was conducted as a narrative synthesis of the current literature on hematologic and immunologic mechanisms in COVID-19 and idiopathic pulmonary fibrosis (IPF). We systematically searched PubMed, Scopus, and Web of Science for peer-reviewed articles published between January 2020 and March 2025. Keywords included combinations of “COVID-19,” “SARS-CoV-2,” “idiopathic pulmonary fibrosis,” “cytokine storm,” “coagulation,” “immune dysregulation,” “endothelial dysfunction,” and “thrombosis”.

We included original research articles (clinical, experimental, and pathological), meta-analyses, and review papers relevant to either disease or their overlap, written in English. Due to the heterogeneity of available studies, we did not perform a formal meta-analysis, and no risk-of-bias assessment was deemed necessary given the narrative nature of this review. A comparative approach was employed to identify overlapping mechanisms of systemic inflammation, immune dysregulation, and fibrogenesis, with the aim of elucidating their potential synergistic role in the pathogenesis and progression of both diseases.

## 3. Hematologic Alterations in COVID-19

The hematological landscape in COVID-19 has emerged as a critical domain of investigation, offering insights into disease pathogenesis, progression, and prognosis. From the early stages of the pandemic, clinicians observed a range of blood-related abnormalities, many of which were predictive of disease severity and clinical outcomes. COVID-19 induces a complex array of hematologic alterations that reflect the interplay between viral pathogenesis, host immune response, and coagulation. Lymphopenia, neutrophilia, thrombocytopenia, hypercoagulability, and cytokine storm are interconnected phenomena that collectively influence disease trajectory.

### 3.1. Lymphopenia, Neutrophilia, and Neutrophil-to-Lymphocyte Ratio (NLR)

Lymphopenia is one of the most consistent hematologic findings in patients with COVID-19, particularly those with severe disease [16]. Studies have shown that more than 80% of hospitalized COVID-19 patients exhibit a reduced lymphocyte count [17]. This lymphocyte depletion primarily affects T cells, including both CD4+ helper and CD8+ cytotoxic subsets [18]. Several mechanisms have been proposed to explain this phenomenon: direct infection of lymphocytes by SARS-CoV-2, cytokine-induced apoptosis, bone marrow suppression, and sequestration of lymphocytes in lymphoid organs or inflamed tissues [19]. Additionally, corticosteroid therapy—frequently administered in moderate to severe COVID-19—may further contribute to lymphopenia and neutrophilia [20,21]. Corticosteroids can induce lymphocyte apoptosis and redistribution, while promoting neutrophil demargination and delayed apoptosis, leading to elevated circulating neutrophil counts [21].

Notably, CD8+ cytotoxic T lymphocytes—which are essential for eliminating virus-infected cells—are both reduced in number and functionally impaired in severe COVID-19 [22]. This impairment includes decreased production of perforin and granzyme B, as well as increased expression of exhaustion markers such as PD-1 and TIM-3 [23,24,25]. These changes correlate with reduced antiviral activity and worse clinical outcomes. Single-cell analyses have also shown limited CD8+ T cell clonal expansion in the lungs, further highlighting the suppression of effective adaptive immunity in severe cases [26].

The depletion of lymphocytes leads to an imbalance in the immune response, impairing the adaptive immunity needed to control viral replication [27]. This immunosuppressed state contributes to prolonged viral shedding and a higher risk of secondary infections [28].

Conversely, neutrophilia is often observed in severe cases of COVID-19 and is indicative of an overactive innate immune response [29]. While neutrophils are essential in the early defense against pathogens, their excessive activation can lead to tissue damage and thromboinflammation [30]. One of the key mechanisms involves the formation of neutrophil extracellular traps (NETs), which, although capable of entrapping pathogens, also promote platelet activation and coagulation cascade initiation [31]. Clinical data further support the pathogenic role of neutrophilia in COVID-19. In a retrospective analysis of 55 patients, neutrophilia was observed in 6 out of 8 individuals with severe disease between days 7 and 19 after symptom onset, paralleling pulmonary lesion progression on chest CT (slope: 0.8; 95% CI: 0.3–1.2). Transcriptomic profiling revealed the upregulation of 17 NET-associated genes in lung tissue and bronchoalveolar lavage fluid, suggesting a direct role of neutrophil activation in lung injury [32]. Similarly, in a Moroccan cohort, neutrophil counts exceeding 7.7 × 10^9^/L on day 5 of hospitalization were identified as independent predictors of ARDS, need for intubation, and mortality—outperforming baseline neutrophil levels, C-reactive protein, and procalcitonin. These findings also showed a positive correlation with IL-6 and respiratory deterioration [33].

In addition to promoting thrombosis, neutrophil extracellular traps (NETs) also contribute directly to endothelial injury. NET components such as histones and neutrophil elastase exert cytotoxic effects on vascular endothelial cells, increasing permeability, disrupting tight junctions, and promoting a prothrombotic and proinflammatory endothelial phenotype [34,35]. This endothelial damage plays a central role in COVID-19-associated microvascular complications and may further exacerbate fibrotic remodeling in predisposed individuals.

The neutrophil-to-lymphocyte ratio (NLR) has emerged as a robust prognostic marker in COVID-19 [36]. A high NLR reflects both inflammation and immunosuppression and has been correlated with poor outcomes, including progression to ARDS and mortality [37]. A systematic review and meta-analysis of 61 studies comprising more than 15,500 patients showed that each unit increase in NLR at hospital admission significantly predicted severe disease (OR 6.22; 95% CI: 4.93–7.84; *p* < 0.001) and all-cause mortality (OR 12.6; 95% CI: 6.88–23.06; *p* < 0.001), with consistent results across low-bias studies and moderate heterogeneity [38]. In a retrospective cohort of 2169 hospitalized COVID-19 patients, elevated NLR and platelet-to-lymphocyte ratio (PLR) values were consistently associated with adverse outcomes—including CPAP use, ICU admission, mechanical ventilation, and death. NLR demonstrated superior prognostic performance (AUROC 0.59–0.81) compared to PLR (AUROC 0.53–0.67) across multiple timepoints [39]. Moreover, recent evidence suggests that NLR also correlates with chest CT severity scores in COVID-19, providing a radiological association that supports its role as a marker of disease burden and pulmonary involvement [40].

### 3.2. Platelet Count and Function

Thrombocytopenia—defined as a platelet count below 150 × 10^9^/L—has been frequently reported in COVID-19 patients, especially those requiring intensive care [41]. While generally mild, a lower platelet count has been associated with increased mortality [42]. The mechanisms behind thrombocytopenia in COVID-19 are multifactorial and include bone marrow suppression, increased peripheral destruction via immune-mediated mechanisms, and increased platelet consumption due to widespread microvascular thrombosis [43]. A recent meta-analysis of 42 patients with COVID-19-associated immune thrombocytopenia (ITP) reported a pooled mean platelet count of 14.5 × 10^9^/L (95% CI: 8.79–20.25), with most cases developing within 2–3 weeks of infection and resolving in under 1 week [44].

Interestingly, despite the presence of thrombocytopenia, COVID-19 is paradoxically associated with a prothrombotic state [45]. Platelets may be activated directly by SARS-CoV-2 or indirectly by inflammatory cytokines, leading to increased aggregation and thrombus formation [46]. Elevated levels of platelet activation markers such as P-selectin and soluble CD40 ligand have been documented, underscoring the role of platelets in COVID-19-associated coagulopathy [47,48]. Experimental models using human angiotensin-converting enzyme 2 (hACE2) transgenic mice have shown that administration of the SARS-CoV-2 spike protein (SP) induces key features of COVID-19-associated coagulopathy (CAC), including endothelial dysfunction, increased transmembrane serine protease 2 (TMPRSS2), neutrophil gelatinase-associated lipocalin (NGAL), matrix metalloproteinases-2 and -9 (MMP-2/9), and reduced disintegrin and metalloproteinase with thrombospondin motifs 13 (ADAMTS13) [49]. These changes suggest a thromboinflammatory process distinct from classical disseminated intravascular coagulation (DIC), characterized by minimal changes in prothrombin time and activated partial thromboplastin time, but significant thrombocytopenia [49].

### 3.3. Coagulation Abnormalities and Hypercoagulability

One of the most striking hematologic features of COVID-19 is the profound dysregulation of the coagulation cascade, manifesting as a hypercoagulable state [50,51]. Elevated D-dimer levels are commonly observed and are strongly associated with worse outcomes. D-dimer, a fibrin degradation product, is a marker of thrombus formation and breakdown, and its rise suggests ongoing coagulation and fibrinolysis [52]. Thrombotic events—including deep vein thrombosis (DVT), pulmonary embolism (PE), and disseminated intravascular coagulation (DIC)—are frequently reported in COVID-19 patients, particularly in critically ill individuals, and have been associated with significantly increased mortality. Studies have proposed various D-dimer cut-off values ranging from 0.5 to over 4 mg/L for predicting PE, with variable sensitivity and specificity [53].

In addition to elevated D-dimer, other abnormalities include prolonged prothrombin time (PT) [54], activated partial thromboplastin time (aPTT) [55], and elevated fibrinogen [56]. In severe cases, disseminated DIC may develop, characterized by systemic activation of coagulation pathways leading to consumption of clotting factors, bleeding, and thrombosis [57].

While elevated D-dimer levels are commonly interpreted as markers of thrombus formation and fibrinolysis, some studies suggest that they may primarily reflect local fibrinolysis resulting from intra-alveolar fibrin degradation during acute lung injury [58].

Microvascular thrombosis, particularly in the pulmonary vasculature, is a key point of severe COVID-19 and contributes significantly to hypoxemia and respiratory failure [59]. Autopsy studies have confirmed widespread microthrombi in lung capillaries, as well as in other organs such as the kidneys and brain [60,61].

The mechanisms underlying COVID-19-induced coagulopathy are multifactorial and include endothelial dysfunction, platelet activation, complement activation, and the release of procoagulant microparticles [51,62]. These processes are further amplified by the systemic inflammatory response and cytokine storm.

### 3.4. Cytokine Storm and Inflammatory Mediators

The cytokine storm refers to a hyperinflammatory state characterized by the excessive release of proinflammatory cytokines, including interleukin-6 (IL-6), tumor necrosis factor-alpha (TNF-α), interleukin-1β (IL-1β), and interferon-gamma (IFN-γ) [63,64]. This exaggerated immune response is a key driver of tissue damage, multiorgan failure, and death in severe COVID-19 [65].

IL-6 plays a central role in the cytokine storm and has been associated with disease severity and poor prognosis [66,67]. Elevated IL-6 levels stimulate the hepatic production of acute-phase reactants such as C-reactive protein (CRP) and fibrinogen, which contribute to the prothrombotic environment [68,69]. TNF-α and IL-1β further propagate inflammation by activating endothelial cells and promoting leukocyte adhesion and transmigration [70].

In addition to their inflammatory effects, cytokines such as IL-6, IL-1β, and TNF-α induce vasodilation and hypotension, which in severe cases may impair organ perfusion or lead to shock [71]. Concurrently, they activate coagulation pathways, enhance platelet aggregation, and increase thrombotic risk.

The cytokine storm also has profound hematologic consequences. It promotes bone marrow suppression, induces lymphocyte apoptosis, and leads to the activation of monocytes and macrophages [72]. These effects contribute to the observed lymphopenia [73], neutrophilia [74], and thrombocytopenia [44]. Moreover, the systemic inflammation activates the coagulation cascade, resulting in the hypercoagulable state discussed earlier [75].

In severe cases, the cytokine storm may resemble secondary hemophagocytic lymphohistiocytosis (sHLH) or macrophage activation syndrome (MAS), characterized by excessive immune cell activation, hyperferritinemia, cytopenias, and multiorgan failure [76,77,78,79]. These hyperinflammatory states have been increasingly recognized in critically ill COVID-19 patients and may represent a form of virus-induced immunopathology requiring targeted immunomodulation.

## 4. IPF and Hematologic Vulnerability

Idiopathic pulmonary fibrosis (IPF) is not only a disease of progressive fibrosis but also a condition characterized by systemic alterations that include hematological and vascular abnormalities [10]. These changes contribute to disease progression and influence susceptibility to secondary complications, such as infections or thromboembolic events [80,81]. The hematologic vulnerabilities observed in IPF are complex and multifactorial, involving endothelial dysfunction, microvascular thrombosis, chronic inflammation, immune dysregulation, and activation of the coagulation cascade [82,83].

### 4.1. Endothelial Dysfunction and Microthrombosis in IPF

Vascular endothelial cells play a critical role in maintaining vascular homeostasis, regulating coagulation, vascular tone, and immune cell trafficking [84]. In IPF, the alveolar epithelium and endothelium are closely interconnected, and injury to one inevitably affects the other. Experimental evidence shows that epithelial damage triggers endothelial dysfunction, basement membrane disruption, and aberrant vascular remodeling, all of which contribute to extracellular matrix accumulation and fibrosis. Vascular signaling pathways—particularly those involving prostanoids and phosphodiesterases—are increasingly recognized as contributors to disease progression [85].

This is thought to result from persistent mechanical stress, oxidative injury, and inflammatory signaling within the fibrotic lung microenvironment [86,87]. Damaged endothelium becomes prothrombotic, proinflammatory, and permeable, creating a vicious cycle that perpetuates both vascular injury and fibrotic remodeling [88]. In the context of COVID-19, elevated levels of circulating endothelial cells have been reported and are considered a marker of ongoing endothelial injury [89]. Their abundance correlates with disease severity, oxygen requirement, and markers of coagulation, supporting the hypothesis that widespread endothelial disruption contributes to both pulmonary and systemic manifestations of COVID-19.

Histological analyses have revealed endothelial cell apoptosis and capillary rarefaction in IPF lungs, along with increased expression of adhesion molecules such as E-selectin, ICAM-1, and VCAM-1, which facilitate leukocyte recruitment and transmigration [90]. For example, findings show that ICAM-1 was weakly expressed in the lung tissue of healthy controls, while E-selectin and VCAM-1 were absent. In IPF, ICAM-1 was broadly upregulated, E-selectin appeared in honeycomb areas, and VCAM-1 was still undetectable [90]. These results suggest E-selectin contributes to neutrophil recruitment in honeycombing, while ICAM-1 may mediate both lymphocyte and neutrophil infiltration [90]. Moreover, studies have reported elevated levels of VCAM-1 in IPF lungs, especially in fibrotic areas and blood vessels, and its levels correlate with worse lung function; TGF-β1 increases VCAM-1 in lung fibroblasts, while VCAM-1 depletion reduces their proliferation by affecting ERK1/2 and cyclin D1 [91]. Thus, VCAM-1 may drive fibroblast proliferation in IPF.

Microvascular thrombosis is a frequent finding in IPF and is believed to contribute to disease progression by exacerbating tissue hypoxia and promoting fibrotic pathways [83]. Inhibition of PAI-1 activity by the specific inhibitor SK-216 reduces the profibrotic effects of TGF-β, including epithelial–mesenchymal transition and fibroblast-to-myofibroblast differentiation, suggesting that PAI-1 acts as a key downstream effector in the pathogenesis of pulmonary fibrosis [92]. Increased plasma levels of plasminogen activator inhibitor-1 are associated with risk of future incident venous thromboembolism [93]. The interplay between thrombosis and fibrosis has led to the hypothesis that IPF may be a prothrombotic state at baseline, independent of acute infections.

### 4.2. Chronic Inflammation and Immune Activation in IPF

Although fibrosis is the defining feature of IPF, chronic inflammation plays an important supportive role in disease pathogenesis. Inflammatory mediators and immune cell infiltrates have been observed in lung tissues from IPF patients, suggesting a continuous, low-grade inflammatory process [94].

Cytokines such as TGF-β, IL-1β, IL-6, IL-10, and TNF-α are upregulated in IPF and contribute to fibroblast activation, epithelial-to-mesenchymal transition, and extracellular matrix deposition [95,96]. In a study, paracrine signaling from fibroblasts derived from patients with idiopathic pulmonary fibrosis was shown to activate the IL-6/STAT3 and TGF-β/Smad3 pathways in healthy lung fibroblasts, contributing to fibrotic progression [97].

Furthermore, various innate immune cells including innate immune populations such as macrophages, neutrophils, fibrocytes, myeloid suppressor cells, and innate lymphoid cells are often activated in IPF lungs, releasing reactive oxygen species, proteases, and additional cytokines that perpetuate inflammation and tissue injury [98]. Macrophages play a dual and dynamic role in the pathogenesis of idiopathic pulmonary fibrosis (IPF), shifting from a proinflammatory M1 phenotype in the early stages—characterized by cytokine release, oxidative stress, and ECM remodeling—to a profibrotic M2 phenotype in advanced disease, marked by the secretion of TGF-β, PDGF, and other mediators that drive fibroblast activation and collagen deposition [99]. This chronic immune activation may also alter the systemic immune response, rendering patients more susceptible to cytokine storm-like phenomena observed in severe COVID-19 [100].

### 4.3. Myeloid Cell Activity and Coagulation Pathways in IPF

Myeloid cells, including monocytes, macrophages, and dendritic cells, are central to the immune dysregulation observed in IPF [101]. These cells serve as key producers of profibrotic and prothrombotic mediators [102,103,104]. In IPF, macrophages exhibit an altered phenotype, skewed toward a profibrotic M2-like polarization, characterized by the secretion of fibrogenic growth factors such as PDGF, TGF-β, and VEGF [105].

Additionally, monocytes from IPF patients may exhibit heightened expression of tissue factor, a potent initiator of the extrinsic coagulation pathway [106]. This contributes to a hypercoagulable state that may be exacerbated by inflammatory cytokines. Coagulation proteases, such as thrombin and factor Xa, can directly activate protease-activated receptors (PARs) on fibroblasts and epithelial cells, promoting fibrotic signaling [107,108].

Recent studies have also identified a link between coagulation and fibrosis through the urokinase-type plasminogen activator (uPA) system [109,110]. Dysregulation of fibrinolysis, driven by elevated levels of PAI-1, leads to impaired matrix turnover and the persistence of fibrotic lesions [111].

Taken together, these findings suggest that IPF is characterized by a baseline hematologic and immune imbalance that may predispose patients to exaggerated responses during systemic infections such as COVID-19. Understanding the hematologic vulnerabilities in IPF provides crucial context for interpreting the impact of superimposed diseases and for identifying potential targets for therapeutic intervention.

**Figure 1:**
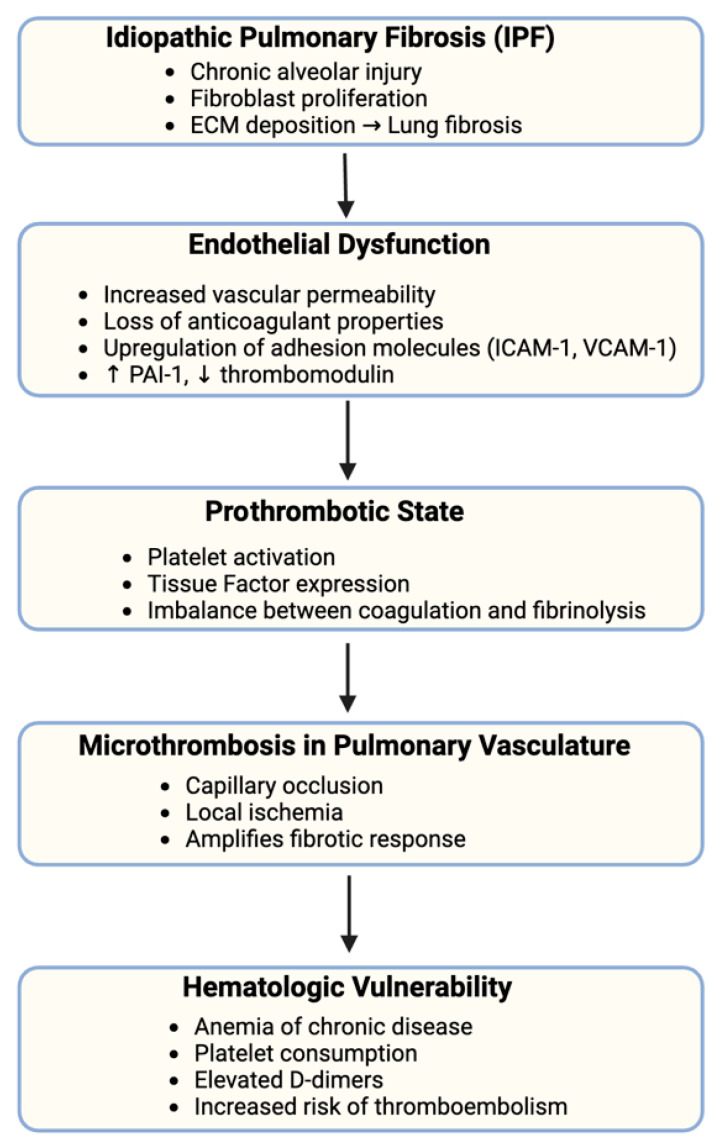
Mechanistic link between idiopathic pulmonary fibrosis and hematologic vulnerability.

Figure 1 illustrates the proposed pathophysiological cascade linking idiopathic pulmonary fibrosis (IPF) to hematologic vulnerability, emphasizing the roles of endothelial dysfunction, prothrombotic shifts, and microvascular thrombosis in disease progression.

## 5. Intersection Between COVID-19 and IPF: Shared Hematologic Mechanisms and Pathological Synergies

The intersection of idiopathic pulmonary fibrosis (IPF) and COVID-19 reveals a series of overlapping hematological and immunopathological mechanisms that may lead to mutual disease amplification. These interactions span from the compounding effects of pulmonary thrombosis to systemic inflammation, immune dysregulation, and cytokine-driven fibrosis.

### 5.1. Worsening of IPF Through COVID-19-Induced Pulmonary Thrombosis

COVID-19 is widely recognized for its capacity to induce a hypercoagulable state and promote pulmonary thrombosis, primarily through endothelial injury, platelet activation, and widespread immune-mediated inflammation [59]. In patients with IPF—who already exhibit microvascular remodeling and a predisposition to thrombosis—the superimposition of SARS-CoV-2 infection can result in widespread microthrombi formation, leading to further alveolar damage, ventilation–perfusion mismatch, and progression of fibrosis [112].

Fibrinogen and its degradation product D-dimer are elevated in severe COVID-19 and are associated with poor prognosis, reflecting both coagulation pathway dysregulation and active involvement in disease progression [113]. Microthrombosis, present in over 90% of fatal COVID-19 cases, results from endothelial injury induced by SARS-CoV-2 and subsequent inflammatory and coagulopathic responses [114]. These events contribute to diffuse alveolar damage (DAD), characterized by epithelial and endothelial injury, neutrophilic infiltration, fibrin deposition, and hypoxemia, ultimately leading to ARDS [114]. Disseminated intravascular coagulation (DIC) further exacerbates fibrin accumulation due to impaired fibrinolysis and anticoagulant pathway disruption [114]. Similarly, in IPF, vascular dysfunction and microthrombosis are known as key fibrotic drivers. Vascular abnormalities in IPF include heterogeneous neovascularization with aberrant vessel dilation and anastomoses, spatial disorganization of endothelial cell populations, impaired endothelial protective signaling (e.g., prostacyclin pathways), and a high prevalence of vascular and metabolic comorbidities [85]. De Rooij et al. demonstrated, via single-nucleus RNA sequencing, a shared enrichment of systemic capillary and venous endothelial cells in COVID-19 and IPF lungs [115]. Their receptor–ligand interaction analysis revealed distinct endothelial communication signatures with non-vascular cells, suggesting that endothelial remodeling and hemostatic imbalance may jointly fuel fibrogenesis through hypoxia-driven and endothelial-to-mesenchymal transition (EndMT)-related pathways, worsening lung stiffness and impairing oxygen exchange [116,117].

### 5.2. Systemic Inflammation and Its Impact on Fibrosis Progression

Both COVID-19 and IPF are marked by heightened systemic inflammation. In IPF, chronic inflammation disrupts the balance of signaling molecules and cellular recruitment, and together with elevated IL-13 and/or TGFβ1, transforms normal healing into a pathogenic fibrotic process [118]. In COVID-19, the systemic inflammatory response can escalate into a cytokine storm, including IL-1, IL-2, IL-6, TNF-α, IFN-γ, IP-10, GM-CSF, MCP-1, and IL-10—some of which correlate strongly with disease severity [119]. This immune dysregulation, marked by an excessive and uncontrolled inflammatory response, contributes significantly to multiorgan damage and poor clinical outcomes in patients with severe COVID-19 [119]. Further, persistent immune dysregulation characterized by elevated proinflammatory and profibrotic cytokines, neutrophilia, and reduced CD28 expression may underlie the development of post-COVID interstitial lung disease [120]. When SARS-CoV-2 infects a patient with underlying IPF, this synergistic inflammatory activation can overwhelm pulmonary tissue, enhance fibroblast activation, and drive excessive extracellular matrix (ECM) deposition.

Studies have shown that circulating levels of IL-6, TNF-α, and IL-1β are significantly elevated in COVID-19 patients with severe disease [121]. These cytokines are also implicated in IPF progression [122]. Their combined presence may contribute to rapid clinical deterioration and a transition from chronic fibrotic disease to acute exacerbation.

### 5.3. Immune Imbalance and Exaggerated Responses to Viral Infection

IPF is characterized by a dysfunctional immune system, including impaired regulatory T cell (Treg) responses, persistent macrophage activation, and elevated neutrophil-to-lymphocyte ratios [123,124]. When infected with SARS-CoV-2, IPF patients may exhibit an amplified immune reaction due to pre-existing dysregulation, resulting in tissue damage, impaired viral clearance, and systemic complications [125].

Alveolar macrophages have a role in mediating lung injury in both IPF and COVID-19, where their overactivation contributes to fibrosis and inflammatory cytokine release. In severe COVID-19, alveolar macrophages promote fibroblast proliferation and fibrosis via the TNFSF12-TNFRSF12A signaling pathway, and silencing TNFRSF12A effectively reverses these profibrotic effects [126]. Monocyte-derived alveolar macrophages exhibit a profibrotic gene expression profile in patients with post-acute COVID-19 respiratory symptoms, with their abundance and associated chemokine levels (CCL2) correlating with the severity of radiographic fibrosis regardless of whether fibrosis was resolving or progressing [127]. In post-COVID lung disease, mechanisms such as epithelial-to-mesenchymal transition, M2 macrophage recruitment, myofibroblast activation, and upregulation of Galectin-1 and -3 mirror key fibrotic pathways seen in idiopathic pulmonary fibrosis, suggesting shared pathogenic processes [128].

### 5.4. Cytokine Expression: TGF-β, IL-6, and TNF-α

The cytokine profiles of COVID-19 and IPF show significant overlap, particularly in the elevation of profibrotic and proinflammatory mediators such as transforming growth factor-beta (TGF-β), interleukin-6 (IL-6), and tumor necrosis factor-alpha (TNF-α).

TGF-β is considered the master regulator of fibrosis. In both IPF and COVID-19, TGF-β drives the differentiation of fibroblasts into myofibroblasts and promotes epithelial–mesenchymal transition (EMT), leading to collagen deposition [129,130]. Bronchoalveolar lavage fluid from COVID-19 patients shows upregulation of TGF-β, correlating with fibrotic radiographic changes. SARS-CoV-2 induces early microthrombosis and immune dysregulation in multiple organs, partly through dysregulated TGF-β1 signaling, which promotes coagulation, immune imbalance, and fibrosis—suggesting that TGF-β inhibitors may offer therapeutic potential in COVID-19-related microvascular complications [131].

IL-6 levels are markedly increased in severe COVID-19 and are also chronically elevated in IPF patients. IPF paracrine signaling in human lung fibroblasts induces IL-6R overexpression, thereby affecting N-HLF survival, while the IL-6/STAT3/Smad3 axis mediates cellular responses that may contribute to fibrotic disease progression [97]. Therapeutic agents like tocilizumab, an IL-6 receptor antagonist, have been trialed in both conditions to mitigate inflammatory damage [97]. IL-6 promotes fibroblast proliferation, endothelial dysfunction, and acute-phase reactant synthesis, contributing to systemic inflammation and thrombosis [132]. In individuals with COVID-19, IL-6 is one of the key inflammatory mediators responsible for triggering the cytokine storm [133].

TNF-α, a central cytokine in both inflammatory and fibrotic pathways, contributes to epithelial apoptosis, reactive oxygen species (ROS) generation, and the activation of NF-κB signaling [134,135]. Elevated TNF-α has been detected in COVID-19-related ARDS and in IPF lung tissue, suggesting that this cytokine plays a shared pathological role [136,137]. Idiopathic pulmonary fibrosis (IPF) fibroblasts secrete TNF-α, which alters the behavior of neighboring normal fibroblasts—promoting NFκB pathway activation—suggesting a self-perpetuating, fibroblast-driven mechanism in IPF pathology [138].

The co-expression and interaction of these cytokines in patients with COVID-19 and underlying IPF may accelerate disease progression, promote acute exacerbations, and increase mortality risk. Targeting these cytokine pathways represents a promising strategy for reducing fibrotic and inflammatory burden in this dual-disease population.

Overall, the convergence of COVID-19 and IPF results in compounded thrombotic, inflammatory, and fibrotic pathology (Table 1).

**Figure 2:**
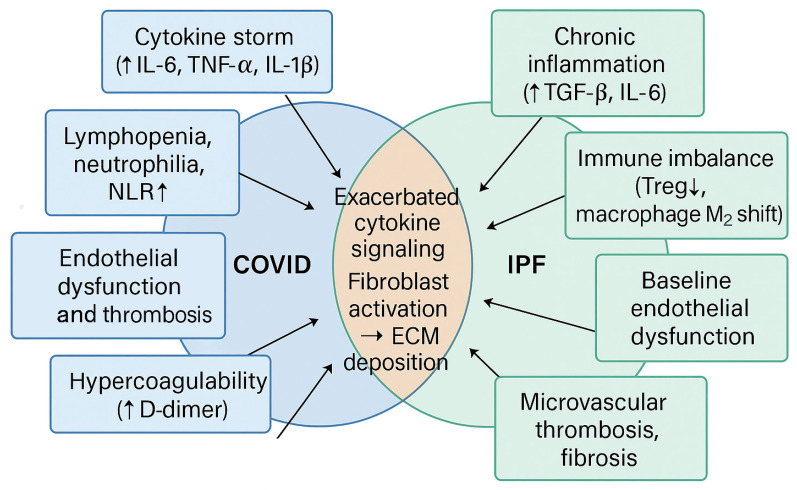
Schematic representation of overlapping mechanisms in COVID-19 and IPF.

As illustrated in Figure 2, both COVID-19 and IPF share a number of overlapping pathophysiological features. These include cytokine-mediated inflammation, endothelial injury, and a prothrombotic state, which may act synergistically to amplify fibrotic progression. Recognizing this mechanistic intersection may support the early identification of high-risk patients and the development of targeted therapeutic strategies.

While the present review highlights multiple hematologic and immunologic mechanisms that may synergistically contribute to disease progression in patients with co-existing IPF and COVID-19, we acknowledge that clinical data on such patient populations are currently limited.

### 5.5. Impact of COVID-19 Vaccination on Hematologic and Fibrotic Pathways

Recent studies have investigated whether COVID-19 vaccination influences hematologic or fibrotic pathways, especially in individuals with pre-existing interstitial lung disease [148]. While transient hematologic changes have been observed post-vaccination—including mild thrombocytopenia or fluctuations in inflammatory markers such as CRP and IL-6—these effects are typically self-limited and not associated with significant clinical deterioration [149]. Importantly, vaccination appears to reduce the severity of SARS-CoV-2 infection and, consequently, the risk of cytokine storm, hypercoagulability, and fibrotic exacerbation [150]. Although COVID-19 vaccination is recommended for patients with IPF, it may rarely trigger acute exacerbations, highlighting the need for close post-vaccination monitoring and further research into potential high-risk clinical phenotypes [151].

## 6. Clinical Implications

The overlapping hematologic disturbances in COVID-19 and idiopathic pulmonary fibrosis (IPF), particularly those involving coagulopathy and endothelial dysfunction, have significant clinical implications. Both conditions are marked by heightened thromboinflammatory responses, characterized by elevated D-dimer, increased neutrophil-to-lymphocyte ratio (NLR), and dysregulated cytokine profiles such as IL-6 and TGF-β, which contribute to microvascular injury and extracellular matrix deposition.

In IPF patients infected with SARS-CoV-2, this convergence may amplify the risk of thromboembolic events, acute exacerbations, and accelerated fibrotic remodeling. Routine monitoring of coagulation and inflammatory markers—including D-dimer, ferritin, and NLR—could help identify early signs of clinical deterioration and guide therapeutic escalation.

These mechanisms also provide a rationale for early antifibrotic intervention, hematologic surveillance, and, in selected cases, targeted anti-cytokine therapy (e.g., IL-6 blockade). Moreover, recognition of this shared pathophysiology underscores the need for structured post-COVID care strategies in IPF patients, including longitudinal lung function follow-up and thromboprophylaxis protocols where appropriate.

A mechanistic understanding of the hematologic and vascular overlap between COVID-19 and IPF thus supports a more personalized and anticipatory approach to clinical management in this highly vulnerable population.

## 7. Conclusions

The intersection between idiopathic pulmonary fibrosis (IPF) and COVID-19 reveals a complex, yet underexplored, pathological interplay with significant clinical implications. This review provides a focused synthesis of the hematologic and immunologic mechanisms shared by both conditions—such as cytokine overexpression (notably TGF-β, IL-6, and TNF-α), endothelial dysfunction, immune dysregulation, and microvascular thrombosis—and emphasizes how SARS-CoV-2 infection may act as a catalyst for accelerated fibrotic remodeling in IPF. Unlike broader analyses of chronic lung disease in the context of viral infections, our aim was to highlight the distinct vulnerability of IPF due to its unique vascular and immune profile. Recognizing these overlapping mechanisms is essential for refining risk stratification in IPF patients, anticipating disease exacerbation during viral infections, and identifying common therapeutic targets. Further research is needed to clarify the long-term impact of SARS-CoV-2 infection in IPF patients, particularly regarding fibrosis progression and acute exacerbations. Prospective clinical studies and experimental models may help identify therapeutic windows for antifibrotic or immunomodulatory interventions in this vulnerable population.

## Figures and Tables

**Table 1:** Pathophysiological intersections between COVID-19 and IPF.

Pathological Aspect	COVID-19	IPF	Combined/Amplified Effects	References
Pulmonary Thrombosis	- Widespread microvascular thrombosis - Elevated D-dimer and fibrinogen - Endothelial injury	- Baseline microvascular remodeling - Prothrombotic endothelial phenotype	Exacerbation of hypoxia, fibrotic progression, increased risk of acute exacerbation	[80]
Systemic Inflammation	- Acute cytokine storm (↑ IL-6, IL-1β, TNF-α) - Intense neutrophilia, hyperinflammation, and neutrophil extracellular traps	- Chronic low-grade inflammation - Persistent release of profibrotic cytokines	Synergistic inflammatory damage, increased fibroblast activity, rapid ECM deposition	[119,139,140,141]
Immune Dysregulation	- Lymphopenia - Neutrophil/monocyte overactivation - Treg cell dysfunction	- Impaired immune tolerance - Chronic macrophage activation - High neutrophil-to-lymphocyte ratio	Exaggerated immune response, delayed viral clearance, enhanced tissue damage	[141,142,143,144,145]
Cytokine Expression (TGF-β, IL-6, TNF-α)	- Upregulation of all three - Associated with severe COVID-19 and ARDS	- Chronically elevated in lung tissue and circulation	Accelerated fibrosis, epithelial–mesenchymal transition (EMT), fibroblast proliferation	[146,147]

## References

[1] Lai C.-C., Shih T.-P., Ko W.-C., Tang H.-J., Hsueh P.-R. (2020). Severe acute respiratory syndrome coronavirus 2 (SARS-CoV-2) and coronavirus disease-2019 (COVID-19): The epidemic and the challenges. Int. J. Antimicrob. Agents.

[2] Zaim S., Chong J.H., Sankaranarayanan V., Harky A. (2020). COVID-19 and Multiorgan Response. Curr. Probl. Cardiol..

[3] Gibson P.G., Qin L., Puah S.H. (2020). COVID-19 acute respiratory distress syndrome (ARDS): Clinical features and differences from typical pre-COVID-19 ARDS. Med. J. Aust..

[4] van Eijk L.E., Binkhorst M., Bourgonje A.R., Offringa A.K., Mulder D.J., Bos E.M., Kolundzic N., Abdulle A.E., van der Voort P.H., Olde Rikkert M.G. (2021). COVID-19: Immunopathology, pathophysiological mechanisms, and treatment options. J. Pathol..

[5] El-Kassas M., Alboraie M., Elbadry M., El Sheemy R., Abdellah M., Afify S., Madkour A., Zaghloul M., Awad A., Wifi M.-N. (2023). Non-pulmonary involvement in COVID-19: A systemic disease rather than a pure respiratory infection. World J. Clin. Cases.

[6] Amoroso D., Bongo S., Copponi A., Rossi V., Di Giorgio R., Bernardini S., Ippoliti L., Morello M. (2025). A Review of the Hematological Picture of Severe COVID-19 Infection. Cureus.

[7] Patrucco F., Solidoro P., Gavelli F., Apostolo D., Bellan M. (2023). Idiopathic Pulmonary Fibrosis and Post-COVID-19 Lung Fibrosis: Links and Risks. Microorganisms.

[8] Bourgonje A.R., Abdulle A.E., Timens W., Hillebrands J.-L., Navis G.J., Gordijn S.J., Bolling M.C., Dijkstra G., Voors A.A., Osterhaus A.D. (2020). Angiotensin-converting enzyme 2 (ACE2), SARS-CoV-2 and the pathophysiology of coronavirus disease 2019 (COVID-19). J. Pathol..

[9] Banu N., Panikar S.S., Leal L.R., Leal A.R. (2020). Protective role of ACE2 and its downregulation in SARS-CoV-2 infection leading to Macrophage Activation Syndrome: Therapeutic implications. Life Sci..

[10] Barratt S.L., Creamer A., Hayton C., Chaudhuri N. (2018). Idiopathic Pulmonary Fibrosis (IPF): An Overview. J. Clin. Med..

[11] Koudstaal T., Wijsenbeek M.S. (2023). Idiopathic pulmonary fibrosis. Presse Médicale.

[12] Hyldgaard C., Møller J., Bendstrup E. (2020). Changes in management of idiopathic pulmonary fibrosis: Impact on disease severity and mortality. Eur. Clin. Respir. J..

[13] Naqvi S.F., Lakhani D.A., Sohail A.H., Maurer J., Sofka S., Sarwari A., Hadi Y.B. (2021). Patients with idiopathic pulmonary fibrosis have poor clinical outcomes with COVID-19 disease: A propensity matched multicentre research network analysis. BMJ Open Resp. Res..

[14] Suri C., Pande B., Sahithi L.S., Sahu T., Verma H.K. (2024). Interplay between Lung Diseases and Viral Infections: A Comprehensive Review. Microorganisms.

[15] Patange A.P., Desai J.V., Pujari B., Marwah A., Dey A. (2024). Dynamic Assessment of Hematological Parameters as Predictive Biomarkers for Disease Severity and Prognosis in COVID-19 Patients: A Longitudinal Study. Cureus.

[16] Terpos E., Ntanasis-Stathopoulos I., Elalamy I., Kastritis E., Sergentanis T.N., Politou M., Psaltopoulou T., Gerotziafas G., Dimopoulos M.A. (2020). Hematological findings and complications of COVID-19. Am. J. Hematol..

[17] Paules C.I., Nordwall J.A., Shaw-Saliba K., Aberg J.A., Gardner E.M., Goodman A.L., Kumarasamy N., Vasudeva S., Vock D.M., North C.M. (2025). Blood absolute lymphocyte count and trajectory are important in understanding severe COVID-19. BMC Infect. Dis..

[18] Kalfaoglu B., Almeida-Santos J., Tye C.A., Satou Y., Ono M. (2021). T-cell dysregulation in COVID-19. Biochem. Biophys. Res. Commun..

[19] Elahi S. (2022). Hematopoietic responses to SARS-CoV-2 infection. Cell Mol. Life Sci..

[20] Lu C., Liu Y., Chen B., Yang H., Hu H., Liu Y., Zhao Y. (2021). Prognostic value of lymphocyte count in severe COVID-19 patients with corticosteroid treatment. Signal Transduct. Target. Ther..

[21] Ronchetti S., Ricci E., Migliorati G., Gentili M., Riccardi C. (2018). How Glucocorticoids Affect the Neutrophil Life. Int. J. Mol. Sci..

[22] Silva M.J.A., Ribeiro L.R., Lima K.V.B., Lima L.N.G.C. (2022). Adaptive immunity to SARS-CoV-2 infection: A systematic review. Front. Immunol..

[23] Rha M.-S., Shin E.-C. (2021). Activation or exhaustion of CD8+ T cells in patients with COVID-19. Cell Mol. Immunol..

[24] Herrmann M., Schulte S., Wildner N.H., Wittner M., Brehm T.T., Ramharter M., Woost R., Lohse A.W., Jacobs T., Schulze Zur Wiesch J. (2020). Analysis of Co-inhibitory Receptor Expression in COVID-19 Infection Compared to Acute Plasmodium falciparum Malaria: LAG-3 and TIM-3 Correlate with T Cell Activation and Course of Disease. Front. Immunol..

[25] Ye C.-H., Hsu W.-L., Peng G.-R., Yu W.-C., Lin W.-C., Hu S., Yu S.-H. (2021). Role of the Immune Microenvironment in SARS-CoV-2 Infection. Cell Transpl..

[26] Notarbartolo S. (2024). T-Cell Immune Responses to SARS-CoV-2 Infection and Vaccination. Vaccines.

[27] Davitt E., Davitt C., Mazer M.B., Areti S.S., Hotchkiss R.S., Remy K.E. (2022). COVID-19 disease and immune dysregulation. Best Pr. Res. Clin. Haematol..

[28] Puhach O., Meyer B., Eckerle I. (2022). SARS-CoV-2 viral load and shedding kinetics. Nat. Rev. Microbiol..

[29] Hazeldine J., Lord J.M. (2021). Neutrophils and COVID-19: Active Participants and Rational Therapeutic Targets. Front. Immunol..

[30] Rong N., Wei X., Liu J. (2024). The Role of Neutrophil in COVID-19: Positive or Negative. J. Innate Immun..

[31] de Bont C.M., Boelens W.C., Pruijn G.J.M. (2019). NETosis, complement, and coagulation: A triangular relationship. Cell Mol. Immunol..

[32] Wang J., Li Q., Yin Y., Zhang Y., Cao Y., Lin X., Huang L., Hoffmann D., Lu M., Qiu Y. (2020). Excessive Neutrophils and Neutrophil Extracellular Traps in COVID-19. Front. Immunol..

[33] El Azhary K., Ghazi B., Kouhen F., El Bakkouri J., Chamlal H., El Ghanmi A., Badou A. (2025). Clinical Impact of Neutrophil Variation on COVID-19 Complications. Diagnostics.

[34] Zuo Y., Yalavarthi S., Shi H., Gockman K., Zuo M., Madison J.A., Blair C., Weber A., Barnes B.J., Egeblad M. (2020). Neutrophil extracellular traps (NETs) as markers of disease severity in COVID-19. JCI Insight.

[35] Middleton E.A., He X.-Y., Denorme F., Campbell R.A., Ng D., Salvatore S.P., Mostyka M., Baxter-Stoltzfus A., Borczuk A.C., Loda M. (2020). Neutrophil extracellular traps contribute to immunothrombosis in COVID-19 acute respiratory distress syndrome. Blood.

[36] Toori K.U., Qureshi M.A., Chaudhry A., Safdar M.F. (2021). Neutrophil to lymphocyte ratio (NLR) in COVID-19: A cheap prognostic marker in a resource constraint setting. Pak. J. Med. Sci..

[37] Basbus L., Lapidus M.I., Martingano I., Puga M.C., Pollán J. (2020). Neutrophil to lymphocyte ratio as a prognostic marker in COVID-19. Medicina.

[38] Ulloque-Badaracco J.R., Ivan Salas-Tello W., Al-kassab-Córdova A., Alarcón-Braga E.A., Benites-Zapata V.A., Maguiña J.L., Hernandez A.V. (2021). Prognostic value of neutrophil-to-lymphocyte ratio in COVID-19 patients: A systematic review and meta-analysis. Int. J. Clin. Pr..

[39] Asperges E., Albi G., Zuccaro V., Sambo M., Pieri T.C., Calia M., Colaneri M., Maiocchi L., Melazzini F., Lasagna A. (2023). Dynamic NLR and PLR in Predicting COVID-19 Severity: A Retrospective Cohort Study. Infect. Dis. Ther..

[40] Todor S.-B., Bîrluțiu V., Topîrcean D., Mihăilă R.-G. (2022). Role of biological markers and CT severity score in predicting mortality in patients with COVID-19: An observational retrospective study. Exp. Ther. Med..

[41] Wool G.D., Miller J.L. (2021). The Impact of COVID-19 Disease on Platelets and Coagulation. Pathobiology.

[42] Yang X., Yang Q., Wang Y., Wu Y., Xu J., Yu Y., Shang Y. (2020). Thrombocytopenia and its association with mortality in patients with COVID-19. J. Thromb. Haemost..

[43] Xu P., Zhou Q., Xu J. (2020). Mechanism of thrombocytopenia in COVID-19 patients. Ann. Hematol..

[44] Alharbi M.G., Alanazi N., Yousef A., Alanazi N., Alotaibi B., Aljurf M., El Fakih R. (2022). COVID-19 associated with immune thrombocytopenia: A systematic review and meta-analysis. Expert. Rev. Hematol..

[45] Semeraro N., Colucci M. (2021). The Prothrombotic State Associated with SARS-CoV-2 Infection: Pathophysiological Aspects. Mediterr. J. Hematol. Infect. Dis..

[46] Sciaudone A., Corkrey H., Humphries F., Koupenova M. (2023). Platelets and SARS-CoV-2 During COVID-19: Immunity, Thrombosis, and Beyond. Circ. Res..

[47] Obeagu E.I., Obeagu G.U., Aja P.M., Okoroiwu G.I.A., Ubosi N.I., Pius T., Ashiru M., Akaba K., Adias T.C. (2024). Soluble platelet selectin and platelets in COVID-19: A multifaceted connection. Ann. Med. Surg..

[48] Agrati C., Sacchi A., Tartaglia E., Vergori A., Gagliardini R., Scarabello A., Bibas M. (2021). The Role of P-Selectin in COVID-19 Coagulopathy: An Updated Review. Int. J. Mol. Sci..

[49] Singh M., Pushpakumar S., Zheng Y., Smolenkova I., Akinterinwa O.E., Luulay B., Tyagi S.C. (2023). Novel mechanism of the COVID-19 associated coagulopathy (CAC) and vascular thromboembolism. NPJ Viruses.

[50] Abou-Ismail M.Y., Diamond A., Kapoor S., Arafah Y., Nayak L. (2020). The hypercoagulable state in COVID-19: Incidence, pathophysiology, and management. Thromb. Res..

[51] Conway E.M., Mackman N., Warren R.Q., Wolberg A.S., Mosnier L.O., Campbell R.A., Gralinski L.E., Rondina M.T., Van De Veerdonk F.L., Hoffmeister K.M. (2022). Understanding COVID-19-associated coagulopathy. Nat. Rev. Immunol..

[52] Nemec H.M., Ferenczy A., Christie B.D., Ashley D.W., Montgomery A. (2022). Correlation of D-dimer and Outcomes in COVID-19 Patients. Am. Surg..

[53] Beidollahkhani S., Fayedeh F., Shoja A., Hassan Nejad E., Hoseinpour M., Fazlpour F., Payandeh A., Pezeshki Rad M., Moodi Ghalibaf A. (2023). d-dimer as a biomarker for COVID-19-associated pulmonary thromboembolism: A narrative review from molecular pathways to the imaging findings. Egypt. J. Bronchol..

[54] Wang L., He W.-B., Yu X.-M., Hu D.-L., Jiang H. (2020). Prolonged prothrombin time at admission predicts poor clinical outcome in COVID-19 patients. World J. Clin. Cases.

[55] Lin J., Yan H., Chen H., He C., Lin C., He H., Zhang S., Shi S., Lin K. (2021). COVID-19 and coagulation dysfunction in adults: A systematic review and meta-analysis. J. Med. Virol..

[56] Ryu J.K., Yan Z., Montano M., Sozmen E.G., Dixit K., Suryawanshi R.K., Matsui Y., Helmy E., Kaushal P., Makanani S.K. (2024). Fibrin drives thromboinflammation and neuropathology in COVID-19. Nature.

[57] Gong F., Zheng X., Zhao S., Liu H., Chen E., Xie R., Li R., Chen Y. (2025). Disseminated intravascular coagulation: Cause, molecular mechanism, diagnosis, and therapy. MedComm.

[58] Hunt B.J., Levi M. (2020). Re The source of elevated plasma D-dimer levels in COVID-19 infection. Br. J. Haematol..

[59] Hanff T.C., Mohareb A.M., Giri J., Cohen J.B., Chirinos J.A. (2020). Thrombosis in COVID-19. Am. J. Hematol..

[60] Wichmann D., Sperhake J.-P., Lütgehetmann M., Steurer S., Edler C., Heinemann A., Heinrich F., Mushumba H., Kniep I., Schröder A.S. (2020). Autopsy Findings and Venous Thromboembolism in Patients with COVID-19: A Prospective Cohort Study. Ann. Intern. Med..

[61] Calabrese F., Pezzuto F., Fortarezza F., Hofman P., Kern I., Panizo A., Von Der Thüsen J., Timofeev S., Gorkiewicz G., Lunardi F. (2020). Pulmonary pathology and COVID-19: Lessons from autopsy. The experience of European Pulmonary Pathologists. Virchows Arch..

[62] Jayarangaiah A., Kariyanna P.T., Chen X., Jayarangaiah A., Kumar A. (2020). COVID-19-Associated Coagulopathy: An Exacerbated Immunothrombosis Response. Clin. Appl. Thromb. Hemost..

[63] Montazersaheb S., Hosseiniyan Khatibi S.M., Hejazi M.S., Tarhriz V., Farjami A., Ghasemian Sorbeni F., Farahzadi R., Ghasemnejad T. (2022). COVID-19 infection: An overview on cytokine storm and related interventions. Virol. J..

[64] Dharra R., Kumar Sharma A., Datta S. (2023). Emerging aspects of cytokine storm in COVID-19: The role of proinflammatory cytokines and therapeutic prospects. Cytokine.

[65] Nazerian Y., Ghasemi M., Yassaghi Y., Nazerian A., Hashemi S.M. (2022). Role of SARS-CoV-2-induced cytokine storm in multi-organ failure: Molecular pathways and potential therapeutic options. Int. Immunopharmacol..

[66] Chen L.Y.C., Hoiland R.L., Stukas S., Wellington C.L., Sekhon M.S. (2020). Confronting the controversy: Interleukin-6 and the COVID-19 cytokine storm syndrome. Eur. Respir. J..

[67] Paranga T.G., Mitu I., Pavel-Tanasa M., Rosu M.F., Miftode I.-L., Constantinescu D., Obreja M., Plesca C.E., Miftode E. (2024). Cytokine Storm in COVID-19: Exploring IL-6 Signaling and Cytokine-Microbiome Interactions as Emerging Therapeutic Approaches. Int. J. Mol. Sci..

[68] Ma L., Willey J. (2022). The interplay between inflammation and thrombosis in COVID-19: Mechanisms, therapeutic strategies, and challenges. Thromb. Update.

[69] Mendes-Filho S.P.D.M., De Souza Pinheiro R., Martins F.S., Giroldi P.J., E Melo R.H., De Oliveira E.L., Dos Santos A.B., Medeiros D.C.O., Lopes J.A., Chaves Y.O. (2024). Kinetics of IL-6, C-reactive Protein and Fibrinogen Levels in COVID-19 Outpatients Who Evolved to Hypoxemia. Clin. Pathol..

[70] Perico L., Benigni A., Casiraghi F., Ng L.F.P., Renia L., Remuzzi G. (2021). Immunity, endothelial injury and complement-induced coagulopathy in COVID-19. Nat. Rev. Nephrol..

[71] Escher R., Breakey N., Lämmle B. (2020). Severe COVID-19 infection associated with endothelial activation. Thromb. Res..

[72] Zeylabi F., Nameh Goshay Fard N., Parsi A., Pezeshki S.M.S. (2023). Bone marrow alterations in COVID-19 infection: The root of hematological problems. Curr. Res. Transl. Med..

[73] Shouman S., El-Kholy N., Hussien A.E., El-Derby A.M., Magdy S., Abou-Shanab A.M., Elmehrath A.O., Abdelwaly A., Helal M., El-Badri N. (2024). SARS-CoV-2-associated lymphopenia: Possible mechanisms and the role of CD147. Cell Commun. Signal.

[74] Reusch N., De Domenico E., Bonaguro L., Schulte-Schrepping J., Baßler K., Schultze J.L., Aschenbrenner A.C. (2021). Neutrophils in COVID-19. Front. Immunol..

[75] Landau N., Shoenfeld Y., Negru L., Segal G. (2022). Exploring the pathways of inflammation and coagulopathy in COVID-19: A narrative tour into a viral rabbit hole. Int. Rev. Immunol..

[76] Hakim N.N., Chi J., Olazagasti C., Liu J.M. (2021). Secondary hemophagocytic lymphohistiocytosis versus cytokine release syndrome in severe COVID-19 patients. Exp. Biol. Med..

[77] Opoka-Winiarska V., Grywalska E., Roliński J. (2020). Could hemophagocytic lymphohistiocytosis be the core issue of severe COVID-19 cases?. BMC Med..

[78] Otsuka R., Seino K. (2020). Macrophage activation syndrome and COVID-19. Inflamm. Regen..

[79] Dong G., Yu J., Gao W., Guo W., Zhu J., Wang T. (2022). Hemophagocytosis, hyper-inflammatory responses, and multiple organ damages in COVID-19-associated hyperferritinemia. Ann. Hematol..

[80] Boonpheng B., Ungprasert P. (2018). Risk of venous thromboembolism in patients with idiopathic pulmonary fibrosis: A systematic review and meta-analysis. Sarcoidosis Vasc. Diffus. Lung Dis..

[81] Lee J.H., Lee H.H., Park H.J., Kim S., Kim Y.-J., Lee J.S., Kim H.C. (2023). Venous thromboembolism in patients with idiopathic pulmonary fibrosis, based on nationwide claim data. Ther. Adv. Respir. Dis..

[82] Magro C.M., Waldman W.J., Knight D.A., Allen J.N., Nadasdy T., Frambach G.E., Ross P., Marsh C.B. (2006). Idiopathic Pulmonary Fibrosis Related to Endothelial Injury and Antiendothelial Cell Antibodies. Hum. Immunol..

[83] Crooks M.G., Hart S.P. (2015). Coagulation and anticoagulation in idiopathic pulmonary fibrosis. Eur. Respir. Rev..

[84] Krüger-Genge A., Blocki A., Franke R.-P., Jung F. (2019). Vascular Endothelial Cell Biology: An Update. Int. J. Mol. Sci..

[85] May J., Mitchell J.A., Jenkins R.G. (2023). Beyond epithelial damage: Vascular and endothelial contributions to idiopathic pulmonary fibrosis. J. Clin. Investig..

[86] Bezerra F.S., Lanzetti M., Nesi R.T., Nagato A.C., Silva C.P.E., Kennedy-Feitosa E., Melo A.C., Cattani-Cavalieri I., Porto L.C., Valenca S.S. (2023). Oxidative Stress and Inflammation in Acute and Chronic Lung Injuries. Antioxidants.

[87] Wang J., Li K., Hao D., Li X., Zhu Y., Yu H., Chen H. (2024). Pulmonary fibrosis: Pathogenesis and therapeutic strategies. MedComm.

[88] Borek I., Birnhuber A., Voelkel N.F., Marsh L.M., Kwapiszewska G. (2023). The vascular perspective on acute and chronic lung disease. J. Clin. Investig..

[89] Guervilly C., Burtey S., Sabatier F., Cauchois R., Lano G., Abdili E., Daviet F., Arnaud L., Brunet P., Hraiech S. (2020). Circulating Endothelial Cells as a Marker of Endothelial Injury in Severe COVID-19. J. Infect. Dis..

[90] Nakao A., Hasegawa Y., Tsuchiya Y., Shimokata K. (1995). Expression of Cell Adhesion Molecules in the Lungs of Patients with Idiopathic Pulmonary Fibrosis. Chest.

[91] Agassandian M., Tedrow J.R., Sembrat J., Kass D.J., Zhang Y., Goncharova E.A., Kaminski N., Mallampalli R.K., Vuga L.J. (2015). VCAM-1 is a TGF-β1 inducible gene upregulated in idiopathic pulmonary fibrosis. Cell Signal.

[92] Omori K., Hattori N., Senoo T., Takayama Y., Masuda T., Nakashima T., Iwamoto H., Fujitaka K., Hamada H., Kohno N. (2016). Inhibition of Plasminogen Activator Inhibitor-1 Attenuates Transforming Growth Factor-β-Dependent Epithelial Mesenchymal Transition and Differentiation of Fibroblasts to Myofibroblasts. PLoS ONE.

[93] Frischmuth T., Hindberg K., Aukrust P., Ueland T., Brækkan S.K., Hansen J., Morelli V.M. (2022). Elevated plasma levels of plasminogen activator inhibitor-1 are associated with risk of future incident venous thromboembolism. J. Thromb. Haemost..

[94] Bringardner B.D., Baran C.P., Eubank T.D., Marsh C.B. (2008). The role of inflammation in the pathogenesis of idiopathic pulmonary fibrosis. Antioxid. Redox Signal.

[95] She Y.X., Yu Q.Y., Tang X.X. (2021). Role of interleukins in the pathogenesis of pulmonary fibrosis. Cell Death Discov..

[96] Bergeron A., Soler P., Kambouchner M., Loiseau P., Milleron B., Valeyre D., Hance A.J., Tazi A. (2003). Cytokine profiles in idiopathic pulmonary fibrosis suggest an important role for TGF-β and IL-10. Eur. Respir. J..

[97] Epstein Shochet G., Brook E., Bardenstein-Wald B., Shitrit D. (2020). TGF-β pathway activation by idiopathic pulmonary fibrosis (IPF) fibroblast derived soluble factors is mediated by IL-6 trans-signaling. Respir. Res..

[98] Ishikawa G., Liu A., Herzog E.L. (2021). Evolving Perspectives on Innate Immune Mechanisms of IPF. Front. Mol. Biosci..

[99] Ge Z., Chen Y., Ma L., Hu F., Xie L. (2024). Macrophage polarization and its impact on idiopathic pulmonary fibrosis. Front. Immunol..

[100] Cilli A., Hanta I., Uzer F., Coskun F., Sevinc C., Deniz P.P., Parlak M., Altunok E., Tertemiz K.C., Ursavas A. (2022). Characteristics and outcomes of COVID-19 patients with IPF: A multi-center retrospective study. Respir. Med. Res..

[101] van Geffen C., Deißler A., Quante M., Renz H., Hartl D., Kolahian S. (2021). Regulatory Immune Cells in Idiopathic Pulmonary Fibrosis: Friends or Foes?. Front. Immunol..

[102] Florez-Sampedro L., Song S., Melgert B.N. (2018). The diversity of myeloid immune cells shaping wound repair and fibrosis in the lung. Regeneration.

[103] Han Z., Liu Q., Li H., Zhang M., You L., Lin Y., Wang K., Gou Q., Wang Z., Zhou S. (2023). The role of monocytes in thrombotic diseases: A review. Front. Cardiovasc. Med..

[104] Rehill A.M., Leon G., McCluskey S., Schoen I., Hernandez-Santana Y., Annett S., Klavina P., Robson T., Curtis A.M., Renné T. (2024). Glycolytic reprogramming fuels myeloid cell-driven hypercoagulability. J. Thromb. Haemost..

[105] Pokhreal D., Crestani B., Helou D.G. (2023). Macrophage Implication in IPF: Updates on Immune, Epigenetic, and Metabolic Pathways. Cells.

[106] van der Poll T. (2008). Tissue factor as an initiator of coagulation and inflammation in the lung. Crit. Care.

[107] D’Alessandro E., Scaf B., Munts C., van Hunnik A., Trevelyan C.J., Verheule S., Spronk H.M.H., Turner N.A., Ten Cate H., Schotten U. (2021). Coagulation Factor Xa Induces Proinflammatory Responses in Cardiac Fibroblasts via Activation of Protease-Activated Receptor-1. Cells.

[108] Posma J.J., Grover S.P., Hisada Y., Owens A.P., Antoniak S., Spronk H.M., Mackman N. (2019). Roles of Coagulation Proteases and PARs (Protease-Activated Receptors) in Mouse Models of Inflammatory Diseases. Arter. Thromb. Vasc. Biol..

[109] Schuliga M., Grainge C., Westall G., Knight D. (2018). The fibrogenic actions of the coagulant and plasminogen activation systems in pulmonary fibrosis. Int. J. Biochem. Cell Biol..

[110] Mercer P.F., Chambers R.C. (2013). Coagulation and coagulation signalling in fibrosis. Biochim. Biophys. Acta (BBA)-Mol. Basis Dis..

[111] Loskutoff D.J., Quigley J.P. (2000). PAI-1, fibrosis, and the elusive provisional fibrin matrix. J. Clin. Investig..

[112] Shen H., Zhang N., Liu Y., Yang X., He Y., Li Q., Shen X., Zhu Y., Yang Y. (2021). The Interaction Between Pulmonary Fibrosis and COVID-19 and the Application of Related Anti-Fibrotic Drugs. Front. Pharmacol..

[113] Kangro K., Wolberg A.S., Flick M.J. (2022). Fibrinogen, Fibrin, and Fibrin Degradation Products in COVID-19. Curr. Drug Targets.

[114] Chen W., Pan J.Y. (2021). Anatomical and Pathological Observation and Analysis of SARS and COVID-19: Microthrombosis Is the Main Cause of Death. Biol. Proced. Online.

[115] De Rooij L.P.M.H., Becker L.M., Teuwen L.-A., Boeckx B., Jansen S., Feys S., Verleden S., Liesenborghs L., Stalder A.K., Libbrecht S. (2023). The pulmonary vasculature in lethal COVID-19 and idiopathic pulmonary fibrosis at single-cell resolution. Cardiovasc. Res..

[116] Nataraj D., Ernst A., Kalluri R. (2010). Idiopathic Pulmonary Fibrosis Is Associated with Endothelial to Mesenchymal Transition. Am. J. Respir. Cell Mol. Biol..

[117] Falleni M., Tosi D., Savi F., Chiumello D., Bulfamante G. (2021). Endothelial-Mesenchymal Transition in COVID-19 lung lesions. Pathol. Res. Pr..

[118] Wilson M.S., Wynn T.A. (2009). Pulmonary fibrosis: Pathogenesis, etiology and regulation. Mucosal Immunol..

[119] Zanza C., Romenskaya T., Manetti A.C., Franceschi F., La Russa R., Bertozzi G., Maiese A., Savioli G., Volonnino G., Longhitano Y. (2022). Cytokine Storm in COVID-19: Immunopathogenesis and Therapy. Medicina.

[120] Oatis D., Herman H., Balta C., Ciceu A., Simon-Repolski E., Mihu A.G., Lepre C.C., Russo M., Trotta M.C., Gravina A.G. (2024). Dynamic shifts in lung cytokine patterns in post-COVID-19 interstitial lung disease patients: A pilot study. Ther. Adv. Chronic Dis..

[121] Hu H., Pan H., Li R., He K., Zhang H., Liu L. (2022). Increased Circulating Cytokines Have a Role in COVID-19 Severity and Death with a More Pronounced Effect in Males: A Systematic Review and Meta-Analysis. Front. Pharmacol..

[122] Kottmann R.M., Hogan C.M., Phipps R.P., Sime P.J. (2009). Determinants of initiation and progression of idiopathic pulmonary fibrosis. Respirology.

[123] Desai O., Winkler J., Minasyan M., Herzog E.L. (2018). The Role of Immune and Inflammatory Cells in Idiopathic Pulmonary Fibrosis. Front. Med..

[124] Ruta V.M., Man A.M., Alexescu T.G., Motoc N.S., Tarmure S., Ungur R.A., Todea D.A., Coste S.C., Valean D., Pop M.C. (2020). Neutrophil-To-Lymphocyte Ratio and Systemic Immune-Inflammation Index-Biomarkers in Interstitial Lung Disease. Medicina.

[125] Buonacera A., Stancanelli B., Colaci M., Malatino L. (2022). Neutrophil to Lymphocyte Ratio: An Emerging Marker of the Relationships between the Immune System and Diseases. Int. J. Mol. Sci..

[126] Guo L., Chen Q., Xu M., Huang J., Ye H. (2024). Communication between alveolar macrophages and fibroblasts via the TNFSF12-TNFRSF12A pathway promotes pulmonary fibrosis in severe COVID-19 patients. J. Transl. Med..

[127] Bailey J.I., Puritz C.H., Senkow K.J., Markov N.S., Diaz E., Jonasson E., Yu Z., Swaminathan S., Lu Z., Fenske S. (2024). Profibrotic monocyte-derived alveolar macrophages are expanded in patients with persistent respiratory symptoms and radiographic abnormalities after COVID-19. Nat. Immunol..

[128] Oatis D., Balta C., Herman H., Ciceu A., Simon-Repolski E., Mihu A.G., Lepre C.C., Russo M., Trotta M.C., D’Amico G. (2025). The interplay between lung galectins and pro-fibrotic markers in post-COVID-19 fibrogenesis: A pilot study. Life Sci..

[129] Fernandez I.E., Eickelberg O. (2012). The Impact of TGF-β on Lung Fibrosis: From Targeting to Biomarkers. Proc. Am. Thorac. Soc..

[130] Alfaro E., Casitas R., Díaz-García E., García-Tovar S., Galera R., Torres-Vargas M., Fernández-Velilla M., López-Fernández C., Añón J.M., Quintana-Díaz M. (2024). TGF-β1 overexpression in severe COVID-19 survivors and its implications for early-phase fibrotic abnormalities and long-term functional impairment. Front. Immunol..

[131] Arguinchona L.M., Zagona-Prizio C., Joyce M.E., Chan E.D., Maloney J.P. (2022). Microvascular significance of TGF-β axis activation in COVID-19. Front. Cardiovasc. Med..

[132] Zhang Y., Zhang Z., Wei R., Miao X., Sun S., Liang G., Chu C., Zhao L., Zhu X., Guo Q. (2020). IL (Interleukin)-6 Contributes to Deep Vein Thrombosis and Is Negatively Regulated by miR-338-5p. Arter. Thromb. Vasc. Biol..

[133] Wang X., Tang G., Liu Y., Zhang L., Chen B., Han Y., Fu Z., Wang L., Hu G., Ma Q. (2022). The role of IL-6 in coronavirus, especially in COVID-19. Front. Pharmacol..

[134] Han D., Ybanez M.D., Ahmadi S., Yeh K., Kaplowitz N. (2009). Redox regulation of tumor necrosis factor signaling. Antioxid. Redox Signal.

[135] Guo Q., Jin Y., Chen X., Ye X., Shen X., Lin M., Zeng C., Zhou T., Zhang J. (2024). NF-κB in biology and targeted therapy: New insights and translational implications. Signal Transduct. Target. Ther..

[136] Mortaz E., Tabarsi P., Jamaati H., Dalil Roofchayee N., Dezfuli N.K., Hashemian S.M., Moniri A., Marjani M., Malekmohammad M., Mansouri D. (2021). Increased Serum Levels of Soluble TNF-α Receptor Is Associated with ICU Mortality in COVID-19 Patients. Front. Immunol..

[137] Lundblad L.K.A., Thompson-Figueroa J., Leclair T., Sullivan M.J., Poynter M.E., Irvin C.G., Bates J.H.T. (2005). Tumor necrosis factor-alpha overexpression in lung disease: A single cause behind a complex phenotype. Am. J. Respir. Crit. Care Med..

[138] Epstein Shochet G., Brook E., Israeli-Shani L., Edelstein E., Shitrit D. (2017). Fibroblast paracrine TNF-α signaling elevates integrin A5 expression in idiopathic pulmonary fibrosis (IPF). Respir. Res..

[139] Faraj S.S., Jalal P.J. (2023). IL1β, IL-6, and TNF-α cytokines cooperate to modulate a complicated medical condition among COVID-19 patients: Case-control study. Ann. Med. Surg..

[140] Borges L., Pithon-Curi T.C., Curi R., Hatanaka E. (2020). COVID-19 and Neutrophils: The Relationship between Hyperinflammation and Neutrophil Extracellular Traps. Mediat. Inflamm..

[141] Heukels P., Moor C.C., Von Der Thüsen J.H., Wijsenbeek M.S., Kool M. (2019). Inflammation and immunity in IPF pathogenesis and treatment. Respir. Med..

[142] Zhao Q., Meng M., Kumar R., Wu Y., Huang J., Deng Y., Weng Z., Yang L. (2020). Lymphopenia is associated with severe coronavirus disease 2019 (COVID-19) infections: A systemic review and meta-analysis. Int. J. Infect. Dis..

[143] McKenna E., Wubben R., Isaza-Correa J.M., Melo A.M., Mhaonaigh A.U., Conlon N., O’Donnell J.S., Ní Cheallaigh C., Hurley T., Stevenson N.J. (2022). Neutrophils in COVID-19: Not Innocent Bystanders. Front. Immunol..

[144] Dhawan M., Rabaan A.A., Alwarthan S., Alhajri M., Halwani M.A., Alshengeti A., Najim M.A., Alwashmi A.S.S., Alshehri A.A., Alshamrani S.A. (2023). Regulatory T Cells (Tregs) and COVID-19: Unveiling the Mechanisms, and Therapeutic Potentialities with a Special Focus on Long COVID. Vaccines.

[145] Achaiah A., Rathnapala A., Pereira A., Bothwell H., Dwivedi K., Barker R., Iotchkova V., Benamore R., Hoyles R.K., Ho L.-P. (2022). Neutrophil lymphocyte ratio as an indicator for disease progression in Idiopathic Pulmonary Fibrosis. BMJ Open Resp. Res..

[146] Silva M.J.A., Ribeiro L.R., Gouveia M.I.M., Marcelino B.D.R., Santos C.S.D., Lima K.V.B., Lima L.N.G.C. (2023). Hyperinflammatory Response in COVID-19: A Systematic Review. Viruses.

[147] Alhamad E.H., Cal J.G., Shakoor Z., Almogren A., AlBoukai A.A. (2013). Cytokine gene polymorphisms and serum cytokine levels in patients with idiopathic pulmonary fibrosis. BMC Med. Genet..

[148] Fukihara J., Kondoh Y. (2023). COVID-19 and interstitial lung diseases: A multifaceted look at the relationship between the two diseases. Respir. Investig..

[149] Ostrowski S.R., Søgaard O.S., Tolstrup M., Stærke N.B., Lundgren J., Østergaard L., Hvas A.-M. (2021). Inflammation and Platelet Activation After COVID-19 Vaccines—Possible Mechanisms Behind Vaccine-Induced Immune Thrombocytopenia and Thrombosis. Front. Immunol..

[150] Jiang Y., Rubin L., Peng T., Liu L., Xing X., Lazarovici P., Zheng W. (2022). Cytokine storm in COVID-19: From viral infection to immune responses, diagnosis and therapy. Int. J. Biol. Sci..

[151] Sgalla G., Magrì T., Lerede M., Comes A., Richeldi L. (2022). COVID-19 Vaccine in Patients with Exacerbation of Idiopathic Pulmonary Fibrosis. Am. J. Respir. Crit. Care Med..

